# High-quality genome assembly and genetic transformation system of *Lasiodiplodia theobromae* strain LTTK16-3, a fungal pathogen of Chinese hickory

**DOI:** 10.1128/spectrum.03311-23

**Published:** 2024-02-13

**Authors:** Tianling Ma, Chenyi Yan, Shuya Zhang, Dong Liang, Chengxin Mao, Chuanqing Zhang

**Affiliations:** 1 Department of Plant Pathology, Zhejiang Agriculture and Forest University, Hangzhou, China; Agroscope, Nyon, Switzerland

**Keywords:** genome, transformation, *Lasiodiplodia theobromae*, tree trunk canker, Chinese hickory

## Abstract

*Lasiodiplodia theobromae,* as one of the causative agents associated with Chinese hickory trunk cankers, has caused huge economic losses to the Chinese hickory industry. Although the biological characteristics of this pathogen and the occurrence pattern of this disease have been well studied, few studies have addressed the related mechanisms due to the poor molecular and genetic study basis of this fungus. In this study, we sequenced and assembled *L. theobromae* strain LTTK16-3, isolated from a Chinese hickory tree (cultivar of Linan) in Linan, Zhejiang province, China. Phylogenetic analysis and comparative genomics analysis presented crucial cues in the prediction of LTTK16-3, which shared similar regulatory mechanisms of transcription, DNA replication, and DNA damage response with the other four Chinese hickory trunk canker-associated *Botryosphaeria* strains including, *Botryosphaeria dothidea*, *Botryosphaeria fabicerciana*, *Botryosphaeria qingyuanensis,* and *Botryosphaeria corticis*. Moreover, it contained 18 strain-specific protein clusters (not conserved in the other *L. theobromae* strains, AM2As and CITRA15), with potential roles in specific host-pathogen interactions during the Chinese hickory infection. Additionally, an efficient system for *L. theobromae* protoplast preparation and polyethylene glycol (PEG) -mediated genetic transformation was firstly established as the foundation for its future mechanisms study. Collectively, the high-quality genome data and the efficient transformation system of *L. theobromae* here set up the possibility of targeted molecular improvements for Chinese hickory canker control.

**IMPORTANCE:**

Fungi with disparate genomic features are physiologically diverse, possessing species-specific survival strategies and environmental adaptation mechanisms. The high-quality genome data and related molecular genetic studies are the basis for revealing the mechanisms behind the physiological traits that are responsible for their environmental fitness. In this study, we sequenced and assembled the LTTK16-3 strain, the genome of *Lasiodiplodia theobromae* first obtained from a diseased Chinese hickory tree (cultivar of Linan) in Linan, Zhejiang province, China. Further phylogenetic analysis and comparative genomics analysis provide crucial cues in the prediction of the proteins with potential roles in specific host-pathogen interactions during the Chinese hickory infection. An efficient PEG-mediated genetic transformation system of *L. theobromae* was established as the foundation for the future mechanisms exploration. The above genetic information and tools set up valuable clues to study *L. theobromae* pathogenesis and assist in Chinese hickory canker control.

## INTRODUCTION

Chinese hickory (*Carya cathayensis* Sarg), known for its high nutritional value and the distinctive fragrance of its nuts, is an economically important Juglandaceae tree native to China (1, 2). At present, there are about 1,300,000 ha of Chinese hickory cultivated in Zhejiang and Anhui provinces, producing an average annual output value of $260 million and a processing output value of $509 million (3). However, nearly 90% of Chinese hickory forests in the above two provinces are seriously affected by the trunk canker disease (4, 5). The symptom of this disease is similar to that of the pecan canker: small, elliptical lesions develop on the bark at the infection sites, which enlarge to form sunken, elongated cankers. These cankers coalesce and form large diffuse areas of blighted tissue, which turn black (1). On Chinese hickory, canker lesions primarily occur on the tree trunk and large branches, but not on leaves, nuts, or panicles (1, 4, 5).

Previously, five Botryosphaeriaceae species have been reported to be associated with Chinese hickory trunk cankers, including *Botryosphaeria dothidea*, *Botryosphaeria fabicerciana*, *Botryosphaeria qingyuanensis*, *Botryosphaeria corticis*, and *Lasiodiplodia theobromae* (6, 7). Notably, *L. theobromae* demonstrated the fastest growth rate, the highest tolerance to high temperatures, and the strongest pathogenicity to Chinese hickory, which might have the potential to become the dominant species (6). *L. theobromae* has several synonyms, including *Botryodiplodia theobromae* Pat. and *Botryosphaeria rhodian* Arx (8, 9). It has a wide geographic distribution as the biotic agent that induces copious necrosis and gummosis, eventually resulting in reduced vigor and lifespan of many economically important woody trees (10, 11), including cacao (12) and citrus (8), besides Chinese hickory trees (6). However, though the biological characteristics and host range of *L. theobromae* have been intensively reported, few studies have focused on the investigation of its survival strategies and pathogenicity mechanisms.

Fungi with disparate genomic features are physiologically diverse possessing species-specific survival strategies and environmental adaptation mechanisms (13). Information initially encoded in the fungal genome is ultimately displayed at the cellular level as functional traits reflecting species-specific life strategies (14, 15). The high-quality genome data and related molecular genetic studies are the basis for revealing the mechanisms behind physiological traits that are responsible for their environmental fitness (16–18). However, among the above-reported Chinese hickory pathogens, the high-quality genome sequence data of four *Botryosphaeria* strains, including *B. dothidea* strain BDLA16-7, *B. cortices* strain BCTK16-35, *B. fabicerciana* strain BFLG18-2, and *B. qingyuanensis* strain BQTK16-30, have been announced (19, 20), but only the genome sequence resource of *L. theobromae* has not been reported. Additionally, transformation protocols for *Lasiodiplodia* species have not been described in detail, and only one research showed an *Agrobacterium tumefaciens*-mediated transformation (ATMT) system for transferring the genes of green fluorescent protein (GFP) and hygromycin B phosphotransferase to *L. theobromae* (21). In consideration of the fact that ATMT systems are tedious to prepare (18, 22), developing a more efficient transformation system for *L. theobromae* is important for further elucidation of its life strategies and pathogenicity mechanisms.

In this study, we sequenced and assembled the LTTK16-3 strain, the genome of *L. theobromae* first obtained from a diseased Chinese hickory tree (cultivar of Linan) in Linan, Zhejiang province, China and established a protoplasmic preparation method and polyethylene glycol (PEG)-mediated genetic transformation system for *L. theobromae*. Additionally, phylogenetic analysis and comparative genomics analysis provide crucial cues in the prediction of the proteins with potential roles in specific host-pathogen interactions during the Chinese hickory infection. Taken together, our study provides information and tools for further exploration of *L. theobromae* survival strategies and pathogenicity mechanisms and sets up the possibility of targeted molecular improvements for Chinese hickory canker control.

## RESULTS

### Genome sequencing, assembly, and annotation

**Fig 1:**
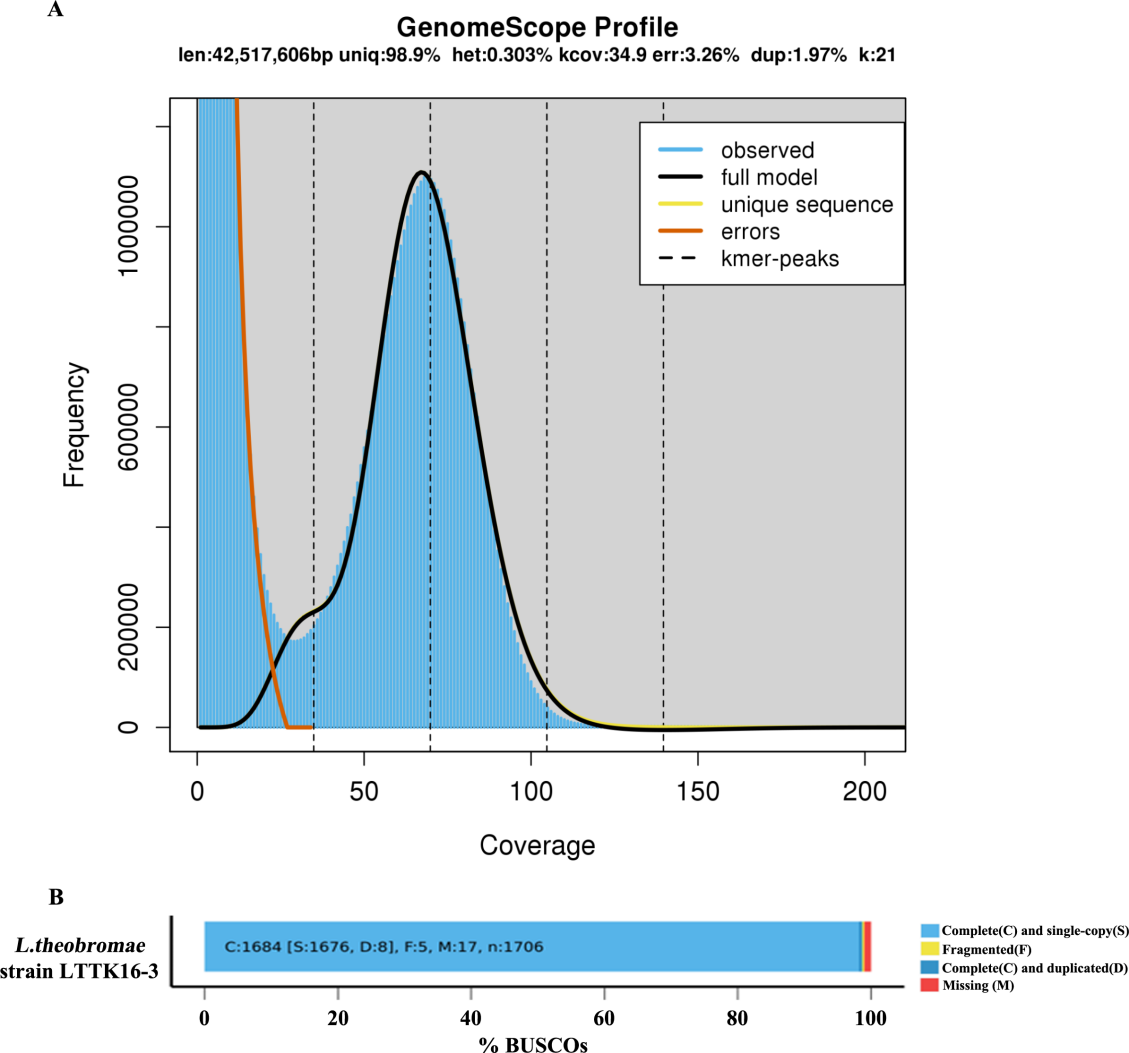
Genome size and heterozygosity estimation by k-mer analysis and the completeness of genome assemblies assessed by BUSCO v5.12. (**A**) The K-mer (*k* = 21) analysis for *Lasiodiplodia theobromae* strain LTTK16-3 revealed the K-mer distributions followed a Poisson distribution with low heterozygosity (<0.5%), and the estimated genome size is 42.52 Mb. (**B**) The completeness of genome assembly of *L. theobromae* strain LTTK16-3 evaluated using BUSCO v5.1.2 in the Ascomycota level revealed the LTTK16-3 strain contains 98.71% complete orthologs at the Ascomycota level (*n* = 1,706).

**Fig 2:**
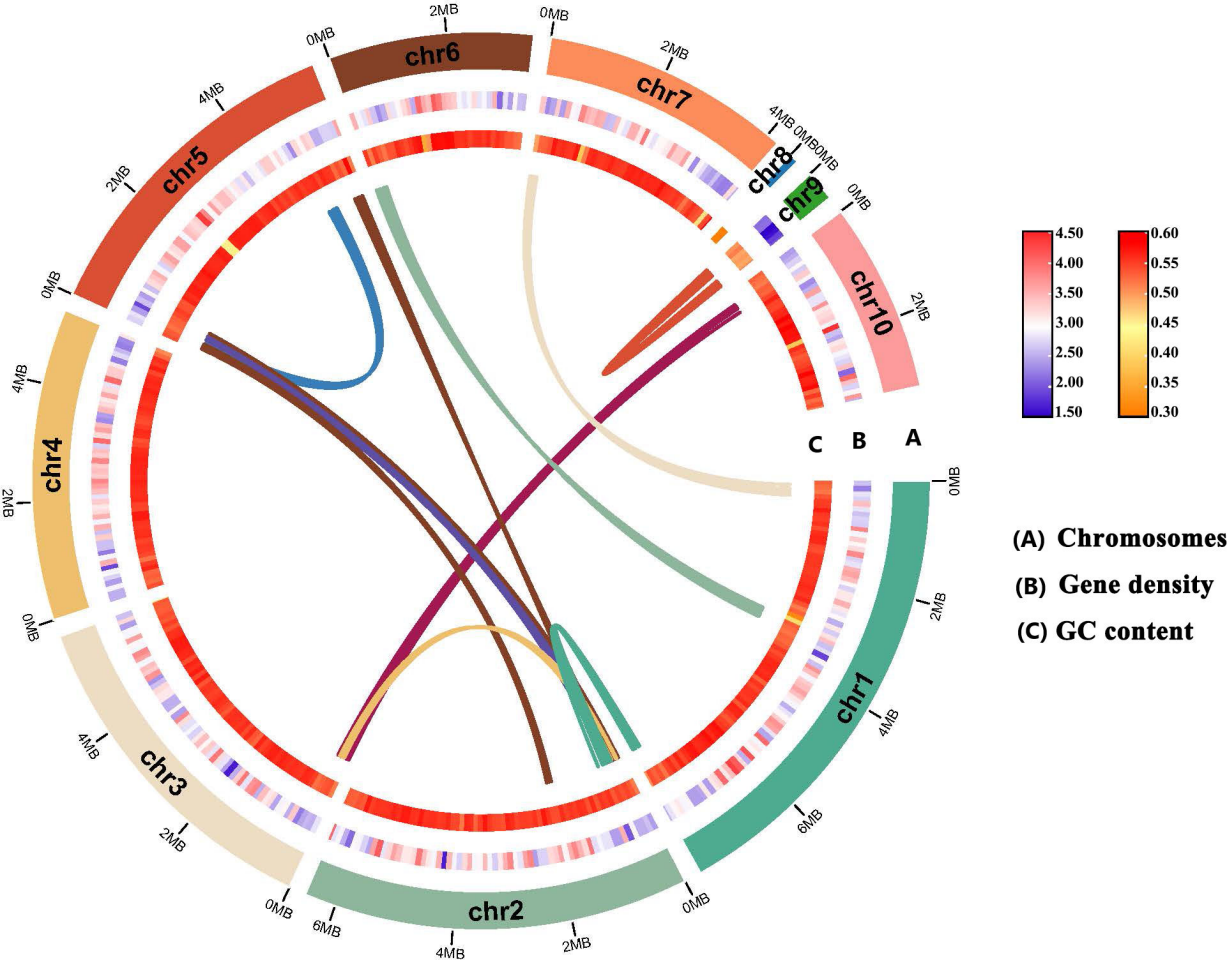
Overview of the *L. theobromae* strain LTTK16-3 genome. The tracks indicate (moving inwards) (A) chromosomes, (B) gene density, and (C) GC content. These density metrics were calculated with 100 kb sliding windows. The syntenic genomic blocks are illustrated with innermost lines.

To generate the basis for studying the origins and mechanisms behind the *L. theobromae* survival strategies and pathogenicity mechanisms, *L. theobromae* strain LTTK16-3, isolated from a Chinese hickory tree (cultivar of Linan) in Linan, Zhejiang province of China (6), was used for genome sequencing. *L. theobromae* has an estimated genome size of 42.52 Mb based on 21 K-mer analysis, and the K-mer distributions followed a Poisson distribution (Fig. 1A). As shown in Table 1, approximately 6 Gb ONT reads were obtained (reads coverage 139×). The *de novo* genome assemblies found that the assembly size of LTTK16-3 strain was 42.82 Mb, the total contig numbers was 10, contig *N*_50_ was 5.67 Mb, and the maximum contig length was 7.93 Mb (Table 1). The completeness of genome assemblies assessed by BUSCO v5.12 (https://busco.ezlab.org/) found that the LTTK16-3 strain contains 98.71% complete orthologs at the Ascomycota level (*n* = 1,706) (Fig. 1B; Table 1). The telomere repeats determined at the start or end region of contigs (5′-TTAGGG-3′/5′-CCCTAA-3′) showed that the assembly of strain LTTK16-3 contained six contigs with (TTAGGG)n start, five contigs with (CCCTAA)n end, and three contigs reached telomere-to-telomere (T2T) chromosomal level (Table 1). Repeats masked before gene prediction found that the repeat content of the LTTK16-3 strain was 3.37% (Table 1; Fig. 1B). The gene prediction showed that a total of 12,516 protein-coding genes were identified in the repeat-masked genome assembly of LTTK16-3 strain (Table 1). Additionally, as shown in Fig. 2, gene density ranged from one to eight genes per 100 kilobases (kb) across the chromosomes, and the GC contents of the total genomes were 54.57% (Table 1; Fig. 2). Intra-genomic syntenic analysis only detected nine syntenic blocks containing 75 pairs of homoeologous genes in the genome of LTTK16-3 strain, which is consistent with its relatively low genomic heterozygosity (Fig. 1A and 2). The gene functional annotation listed in Table 1 shows that the LTTK16-3 strain contained around 2,457 pathogen-host interaction genes, 237 carbohydrate-active enzymes, and 190 cytochrome P450 enzymes. Additionally, a total of 715 putative secreted proteins were identified using our previously defined pipeline (Table 1) (23), and 51 secondary metabolite biosynthetic genes were identified using online antiSMASH (https://fungismash.secondarymetabolites.org/#!/start) (Table 1) (24, 25).

**TABLE 1:** Genome features of Chinese hickory canker causative agent *Lasiodiplodia theobromae* (strain LTTK16-3)

Features	*L. theobromae* (strain LTTK16-3)
GWH accession^*a*^	GWHBFSF00000000
ONT reads (Gb)	5.96
Reads coverage (×)	139
Assembly size (Mb)	42.82
Contig number	10
Contig *N*_50_ (Mb)	5.67
Contig *L*_50_	4
Maximum contig length (Mb)	7.93
Telomeric repeats (TTAGGG)n^*b*^	6/5:3
GC content	54.57%
Repeat sequences	3.37%
BUSCO completeness	98.71%
Protein-coding genes	12,516
Pathogen-host interaction genes	2,457
Carbohydrate active enzymes	237
Cytochrome P450 enzymes	190
Putative secreted proteins	715
SMBGCs^*c*^	51

^*a*^ GWH the Genome Warehouse, https://ngdc.cncb.ac.cn/gwh.

^*b*^ Number of contigs with telomeric repeats at 5′ start/3′ end: both.

^*c*^ SMBGCs (secondary metabolite biosynthesis gene clusters) analyzed by the fungal version of antiSMASH v5.2.0.

### Phylogenetic analysis and comparative genomics analysis

**Fig 3:**
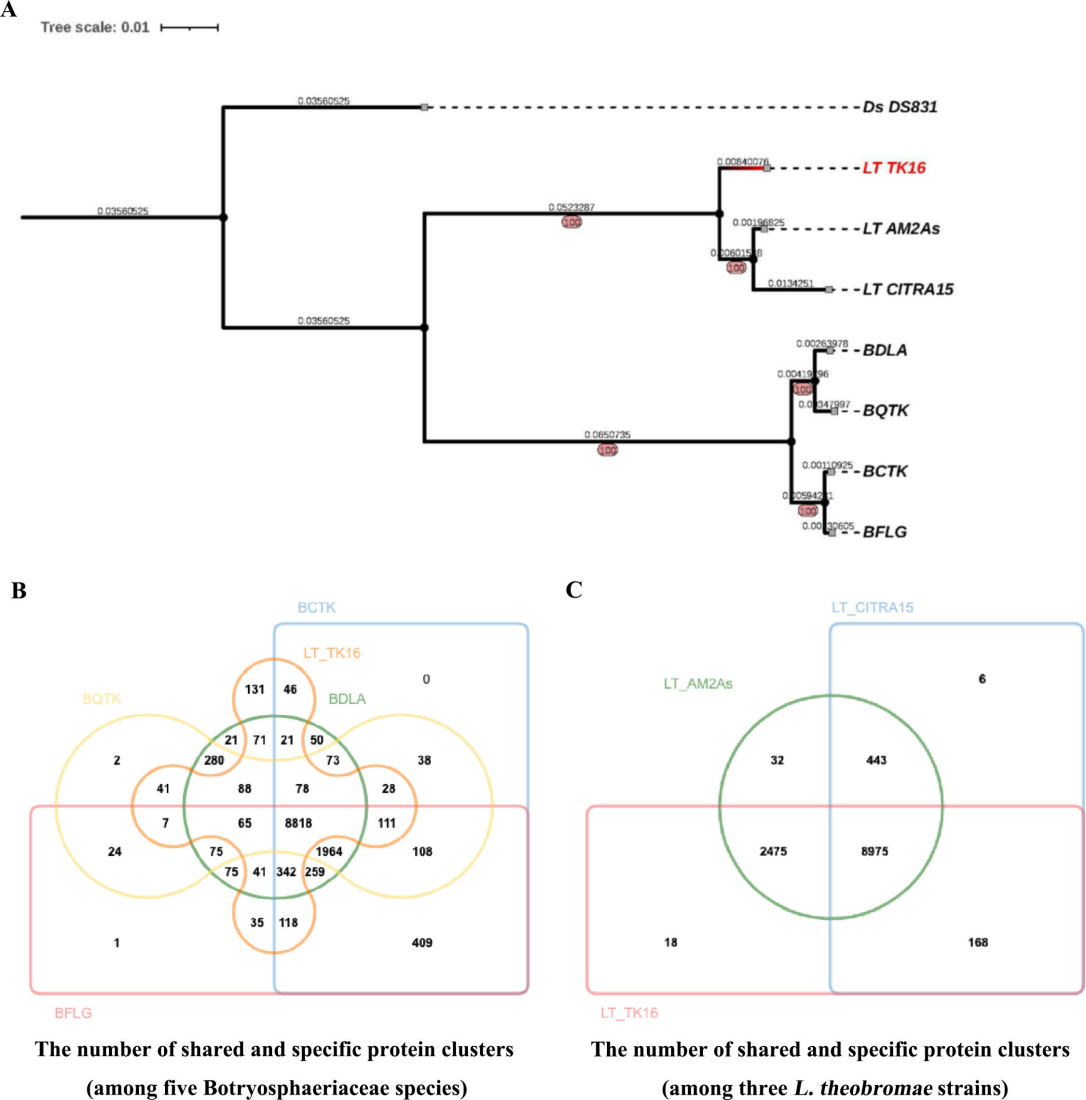
Phylogenetic analysis and comparative genomics analysis of *L. theobromae* strain LTTK16-3. (**A**) The phylogenetic tree of the LTTK16-3 strain. The species tree was inferred as part of the OrthoFinder pipeline using the STAG algorithm and rooted by STRIDE. STRIDE probability values are shown at internal nodes. Scale bar indicates the number of substitutions per site. (**B**) Venn diagram depicting the number of shared and specific protein clusters among five Botryosphaeriaceae species. (**C**) Venn diagram depicting the number of shared and specific protein clusters among three *L. theobromae* strains.

The phylogenetic tree of the LTTK16-3 strain was made based on the result of orthogroups using the Species Tree inference from All Genes (STAG) and Species Tree Root Inference from gene Duplication Events (STRIDE) in OrthoFinder software (26). As shown in Fig. 3A, the phylogenetic tree revealed that LTTK16-3 clustered with reported *L. theobromae* strain CITRA15 isolated from citrus and *L. theobromae* strain AM2As isolated from cacao, whereas the other four Chinese hickory trunk canker-associated *Botryosphaeria* species, including *B. dothidea*, *B. fabicerciana*, *B. qingyuanensis,* and *B. cortices* formed a single monophyletic clade individually, indicating that among these Chinese hickory trunk canker causative agents, LTTK16-3 strain was genetically far away from the other four *Botryosphaeria* strains. Consistently, orthologous protein cluster analysis conducted at the online web service OrthoVenn2 (https://orthovenn2.bioinfotoolkits.net/home) found that LTTK16-3 contained the most species-specific protein clusters, i.e., 131, while the other four *Botryosphaeria* strains only contained 0–21 species-specific protein clusters (Fig. 3B). Additionally, these five Chinese hickory trunk canker causative fungi shared 8,818 core orthologous protein clusters (including 9,095 orthologous proteins) (Fig. 3B), and a Kyoto Encyclopedia of Genes and Genomes (KEGG) analysis mapped above 8,818 core orthologous protein clusters to 137 KEGG pathways (Table S2) with seven significantly enriched KEGG pathways (*P <* 0.05), including the ribosome, basal transcription factor, RNA polymerase, nucleotide excision repair, RNA degradation, DNA replication, and mismatch repair (Fig. S1), which indicates the similar regulatory mechanisms of transcription, DNA replication, and DNA damage response among the five Chinese hickory trunk canker causative pathogens. Intriguingly, comparative analysis between LTTK16 and other previously reported *L. theobromae* strains, including AM2As and CITRA15 (*E*-value cutoff is 1e-05 and inflation value cutoff is 2.0) showed that a total of 8,975 orthogroups were shared by LTTK16, AM2As, and CITRA15, while 18, 32, and 6 orthogroups were found to be specific to LTTK16, AM2As, and CITRA15, respectively (Fig. 3C). Additionally, bidirectional BLAST analysis found that most (72%) of the above 18 LTTK16-3 strain-specific protein clusters (including 39 LTTK16-3 strain-specific proteins) have orthologous proteins in the other four Chinese hickory trunk canker-related *Botryosphaeria* strains (Table S3). Further analysis of above the LTTK16-3 strain-specific proteins by GO (Table S4) and Pfam domain annotation (Table S5) found two specific kinases, LT_TK16_09351 and LT_TK16_09352, containing the Pkinase domain (PF00069) enriched in the GO term “intracellular signal transduction” (GO:0035556), which might contribute to the specific survival strategies of Chinese hickory pathogens.

### Sensitivity of *L. theobromae* to geneticin (G-418) and protoplast Preparation

**Fig 4:**
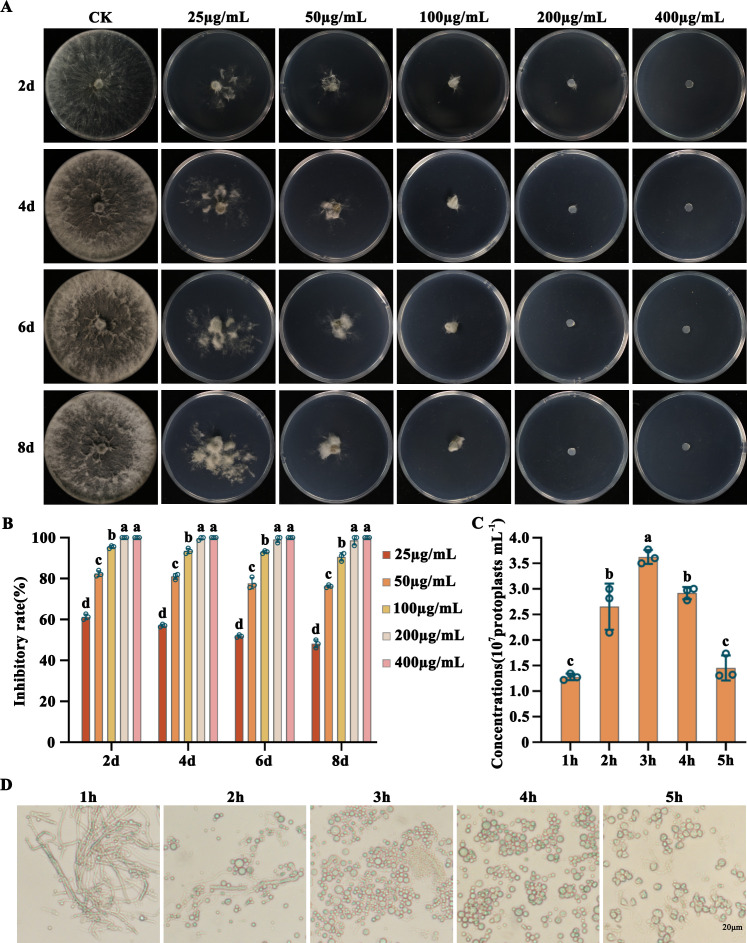
Determination of the sensitivity of *L. theobromae* to G-418 and the preparation of protoplasts. (**A**) Sensitivity of *L. theobromae* to G-418. *L. theobromae* was cultured on PDA plates with 0 (CK), 25, 50, 100, 200, and 400 µg/mL of G-418. *L. theobromae* could grow when the concentration of G-418 was less than 100 µg/mL but failed to grow when the concentration of G-418 was more than 200 µg/mL. The images were taken after 2-, 4-, 6-, and 8-day incubation at 25°C, and the mycelial growth inhibition was calculated for each concentration. (**B**) The mycelial growth inhibition of *L. theobromae* under the above concentrations of G-418 treatments. (**C**) Numbers of *L. theobromae* protoplasts at different digestion times in protoplast preparation. The mycelia were digested in enzymatic digestion solution at 30°C for 1, 2, 3, 4, and 5 h. Sufficient protoplasts were formed at 3 h, with concentrations reaching 3.62 ± 0.14 × 10^7^ protoplasts mL^−1^. (**D**) Changes in the number and quality of protoplasts at different digestion times. Bar: 20 µm. For panels B and C, the mean and standard deviation were estimated with data from three independent biological replicates (*n* = 3). Different letters indicate significant differences based on ANOVA followed by Tukey’s multiple comparisons test (*P* < 0.05).

To establish a transformation system for *L. theobromae*, the sensitivity to G-418 was first conduced for subsequent selection of transformants carrying the functional G-418 resistant gene. The sensitivity of the wild-type *L. theobromae* strain LTTK16-40 was examined on potato dextrose agar (PDA) medium containing G-418 at concentrations ranging from 0 to 400 µg/mL. As shown in Fig. 4, ≥100 µg/mL of G-418 was able to significantly inhibit the LTTK16-40 growth, and ≥200 µg/mL of G-418 completely inhibited the LTTK16-40 growth. Therefore, successful transformants carrying the functional G-418 resistant gene were screened using PDA with 200 µg/mL of G-418 (Fig. 4A and B).

To screen the best enzymatic digestion condition for protoplast preparation during the PEG-mediated protoplast transformation, the prepared fresh mycelia were placed in 10 mL of enzymatic digestion solution (0.3 g cellulase, 0.3 g lysozyme, 0.25 g lysing enzyme, and 0.08 g driselase) and shaken at 30°C, 100 rpm. The protoplasts began to form after 0.5 h of enzymatic treatment (Fig. S2). The amount of protoplasm production increased continuously during the first 3 h and then leveled off. After 4-h of enzymatic digestion, the quality of protoplasm production started to decrease, and the cell membranes of some protoplasm ruptured with irregular shape (Fig. 4C and D). Therefore, it was proved that the suitable enzymatic digestion time for *L. theobromae* protoplast preparation during transformation was 3–4 h (Fig. 4C and D).

### Screening and detection of transformants

**Fig 5:**
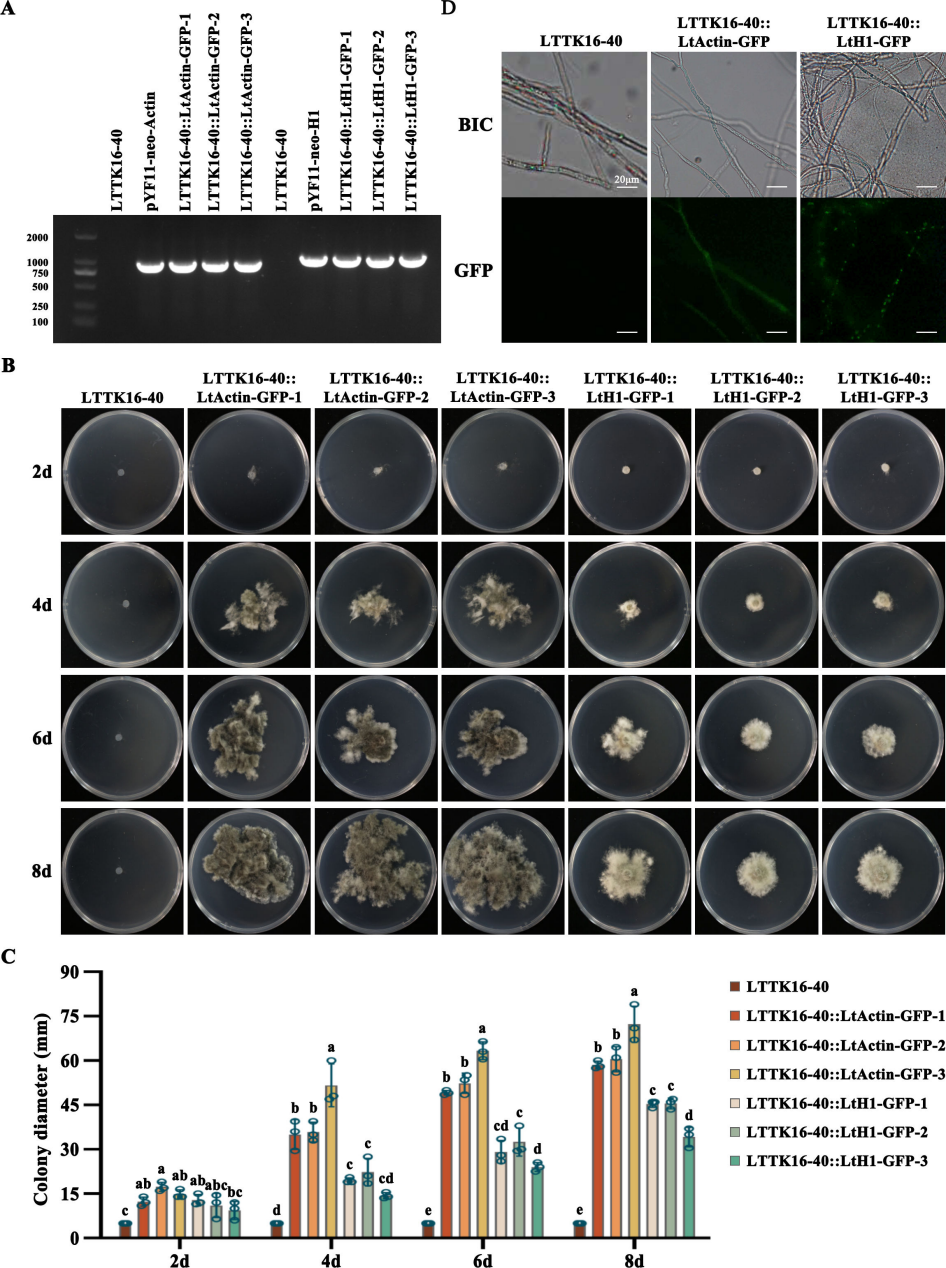
Screening and detection of transformants. (**A**) PCR identification of transformants. The agarose gel electrophoresis showed the primers Actin-GFP-ID-F/GFP-ID-R and H1-GFP-ID-F/GFP-ID-R could give an amplicon around 800 bp for the plasmids and the transformants, including pYF11-neo-Actin, pYF11-neo-H1, LTTK16-40::LtActin-GFP-1-3, and LTTK16-40::LtH1-GFP-1-3 but not for wild type (LTTK16-40). (**B**) Transformants’ selection using PDA selection medium containing 200 µg/mL G-418. The transformant strains (LTTK16-40::LtActin-GFP-1-3 and LTTK16-40::LtH1-GFP-1-3) could grow on PDA plates, while the wild-type LTTK16-40 failed to grow. The images were taken after 2-, 4-, 6-, and 8-day incubation at 25°C. (**C**) Colony diameters of LTTK16-40, LTTK16-40::LtActin-GFP-1-3, and LTTK16-40::LtH1-GFP-1-3 on PDA plates with 200 µg/mL of G-418 after 2-, 4-, 6-, and 8-day incubation at 25°C. Mean and standard deviation were estimated with the data from three independent biological replicates (*n* = 3). Different letters indicate significant differences based on ANOVA followed by Tukey’s multiple comparisons test (*P* < 0.05). (**D**) Confocal microscopic examination of transformants. The figures above are the mycelia of wild type (LTTK16-40) and transformants (LTTK16-40::LtActin-GFP and LTTK16-40::LtH1-GFP) in visible light; the figures below show that the green fluorescence was observed in the mycelia of transformants, using a confocal microscope. Bars: 20 µm.

**Fig 6:**
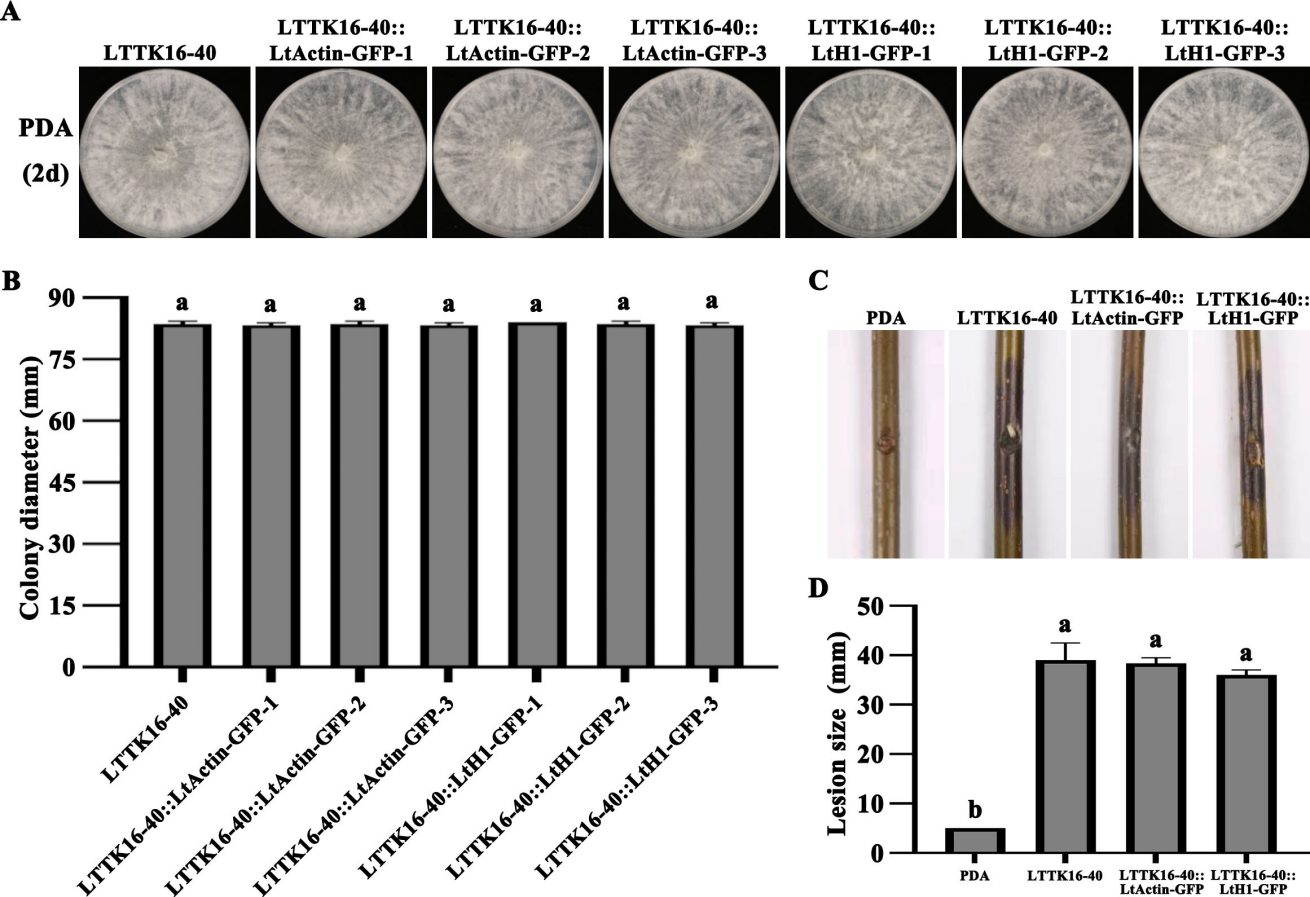
Growth and pathogenicity examination. (**A**) Colony morphology of wild-type strain (LTTK16-40) and transformant strains (LTTK16-40::LtActin-GFP and LTTK16-40::LtH1-GFP) on PDA media for 2 days. (**B**) Colony diameters of wild-type strain (LTTK16-40) and transformant strains (LTTK16-40::LtActin-GFP and LTTK16-40::LtH1-GFP). The mean and standard deviation were estimated with the data from three independent biological replicates (*n* = 3). Different letters indicate significant differences based on ANOVA followed by Tukey’s multiple comparisons test (*P* < 0.05). (**C**) Pathogenicity detection of the transformants. Symptoms on the branch of Chinese hickory after wound-inoculating mycelia of wild-type strain (LTTK16-40) and transformant strains (LTTK16-40::LtActin-GFP and LTTK16-40::LtH1-GFP), respectively, for 4 days. CK: PDA was used as a control. (**D**) Lesion size on Chinese hickory branches caused by wild-type strain (LTTK16-40) and transformant strains (LTTK16-40::LtActin-GFP and LTTK16-40::LtH1-GFP). CK: PDA was used as a control. The mean and standard deviation were estimated with the data from three independent replicates (*n* = 3). Different letters indicate significant differences based on ANOVA followed by Tukey’s multiple comparisons test (*P* < 0.05).

The two recombinant pYF11-neo plasmids containing LtActin-GFP fusion cassette and LtH1-GFP fusion cassette were transferred into the wild-type *L. theobromae* strain LTTK16-40, named LTTK16-40:: LtActin-GFP and LTTK16-40::LtH1-GFP, respectively. The corresponding transformants were selected using PDA selection medium containing 200 µg/mL G-418 and verified by PCR identification together with sequencing to ensure the accuracy of the in-frame fusion region (Fig. 5A through C). As shown in Fig. 5B and C, the confirmed transformants were able to grow on PDA selection medium, while the wild-type *L. theobromae* strain LTTK16-40 was unable to grow. Additionally, further confocal microscopic examination showed that the above transformants exhibited intense fluorescence, suggesting that the designed PEG-mediated protoplast transformation system in this study works successfully for *L. theobromae* transformation (Fig. 5D). The recombinant pYF11-neo plasmids containing GFP-fused genes were successfully transferred into LTTK16-40 via this system, and the corresponding GFP-fused protein could stably express in *L. theobromae* (Fig. 5D). Additionally, growth and pathogenicity examination found that the growth rate and virulence of transformants LTTK16-40:: LtActin-GFP and LTTK16-40::LtH1-GFP demonstrated no significant differences to that of the wild-type *L. theobromae* strain LTTK16-40 (Fig. 6), indicating this transformation system had no side effect on *L. theobromae* growth and virulence.

## DISCUSSION

As one of the causative agents associated with Chinese hickory trunk cankers, *L. theobromae* has caused huge economic losses to the Chinese hickory industry due to its extremely strong virulence (6). Besides damaging Chinese hickory trees, this pathogen is becoming a significant threat to various woody plants with wide geographic distribution (8, 9, 12). Here, we sequenced and assembled the LTTK16-3 strain, the genome of *L. theobromae* first obtained from a diseased Chinese hickory tree (cultivar of Linan) in Linan, Zhejiang province of China. The phylogenetic tree revealed that among the five Chinese hickory trunk canker causative agents, the LTTK16-3 strain was genetically far away from the other four *Botryosphaeria* strains. Comparing the genomic information of *L. theobromae* strain LTTK16-3 with the published genomes of the other four Chinese hickory trunk canker-associated *Botryosphaeria* species (19, 20) found that the genome assembly of strain LTTK16-3 had the minimum assembly size and contig number, as well as the largest contig *N*_50_ and the maximum contig length (Table 1). Additionally, the repeat contents of strain LTTK16-3 (3.37%) were the smallest one (3.37%) compared to that of the other four reported Chinese hickory canker causal agents, *B. dothidea* (BDLA16-7 strain, 7.96%), *B. cortices* (BCTK16-35 strain, 8.50%), *B. fabicerciana* (BFLG18-2 strain, 8.20%), and *B. qingyuanensis* (BQTK16-30 strain, 6.03%) (19, 20). The orthologous protein cluster analysis found that LTTK16-3 contained 131 species-specific protein clusters that are not conserved among other Chinese hickory trunk canker causative *Botryosphaeria* species (Fig. 3B). The difference initially appeared in genomics is ultimately displayed at the cellular level as disparate strategies for survival and infection. Our previous study has reported that *L. theobromae* was the most virulent pathogen of Chinese hickory among the Botryosphaeriaceae species (6). Thus, we speculated that the above species-specific protein clusters identified here likely support that discrepancy in virulence.

In fungi, protein kinases (PKs), as well as the reversible protein phosphorylation, play critical roles in signal transduction, growth, sexual reproduction, and stress responses (27, 28). For instance, *Fusarium graminearum,* a devastating fungal pathogen of cereal crops, has 116 PK genes, of which 20 of them appear to be essential, 42 of them are important for virulence, 32 of them are associated with vegetative growth, and 20 of them play important roles in sexual production, and the corresponding mutants fail to produce perithecia (28). Additionally, PKs, such as FgYak1, FgSsk2, FgPbs2, and FgHog1, are the key regulators in host-derived oxidative stress response during the infection (28). The kinase FgCak1 regulating the phosphorylation of FgNem1 at Thr72, Ser187, and Ser287 together with kinase FgTor and phosphatase FgPpg1 under the target of rapamycin pathway plays crucial roles in *F. graminearum* lipid droplet biogenesis, vegetative growth, sexual development, and virulence (29). In *Botrytis cinerea,* a fungal pathogen causing pre- and post-harvest diseases in more than 200 economically important crops, the mitogen-activated protein kinase BcMkk1 responsible for orchestrating changes of fungal cell wall positively regulates pathogenicity via the cell wall integrity pathway, which controls vegetative growth, conidiation, and responses to cell wall and oxidative stresses (30). Thus, the PKs with specific functions contribute to the related infection and survival strategies of fungal pathogens (28–30). In the *L. theobromae* strain LTTK16-3, there are two predicted strain-specific PKs, LT_TK16_09351 and LT_TK16_09352, that are not conserved in the other *L. theobromae* strains (AM2As and CITRA15) and potentially involved in intracellular signal transduction. Therefore, investigating the potential functions of the above two predicted PKs could be a valuable way to further explore the mechanisms involved in the specific host-pathogen interactions during the Chinese hickory infection.

The development of genetic transformation techniques is a precondition for gene function and related mechanism analysis (31, 32). In filamentous fungi, the PEG-mediated genetic transformation system has been widely demonstrated to be a simple and effective system for transformation (33, 34). PEG is a crucial agent enhancing transformation efficiency (35). It can promote adhesion by forming the molecular bridge between cytomembrane and exogenous nucleic acids (18). Additionally, it can also alter the membrane permeability and facilitate the entry of DNA into cells by inducing disordered charges on the cytomembrane surface (18). The PEG-mediated genetic transformation mainly contains two steps, including protoplast preparation and PEG-mediated fusion of exogenous nucleic acids and protoplasts. Due to the high variety of the fungal cell wall components among different species (18), there is no universal transformation method that can be applied to different fungal strains. Here, we first established a PEG-mediated genetic transformation system with an efficient protoplast preparation protocol for *L. theobromae*. Compared to the ATMT system for *L. theobromae* transformation, which employs pycnidiospores as transformation targets (21), the PEG-mediated genetic transformation system here directly targets fungal protoplasts. Yet, sporulation is a difficult task, and sporulation efficiency can be negatively compromised by temperature, light, and substrate regimes (21). Thus, the employment of protoplasts, rather than pycnidiospores, as the biological target in transformation is more appropriate, which is an improvement in advancing the transformation efficiency.

Overall, here we sequenced and assembled the LTTK16-3 strain, the genome of *L. theobromae* first obtained from a diseased Chinese hickory tree (cultivar of Linan) in Linan, Zhejiang province of China (Fig. 1 and 2). Phylogenetic analysis and comparative genomics analysis found that though *L. theobromae* LTTK16-3 strain was genetically far away from the other four *Botryosphaeria* strains, all five Chinese hickory trunk canker causative pathogens shared 8,818 core orthologous protein clusters (Fig. 3). KEGG pathway analysis of the above core orthologous protein clusters presented crucial cues in the prediction of the similar regulatory mechanisms of transcription, DNA replication, and DNA damage response among five pathogens (Fig. S1; Table S2). Orthologous protein cluster analysis of LTTK16 and other previously reported *L. theobromae* strains AM2As and CITRA15 showed that LTTK16-3 strain contained 18 LTTK16-3 strain-specific protein clusters (including 39 LTTK16-3 strain-specific proteins), and bidirectional BLAST analysis found that 72% of the above strain-specific proteins have orthologous proteins in the other four Chinese hickory trunk canker related*-Botryosphaeria* strains (Table S3), indicating their potential roles in specific host-pathogen interactions during the Chinese hickory infection. Thus, we developed an efficient PEG-mediated genetic transformation system establishing the foundation for future studies on the mechanisms of *L. theobromae* and setting up the possibility of targeted molecular improvements for Chinese hickory canker control.

## MATERIALS AND METHODS

### Fungal strain, culture conditions, and geneticin (G-418) sensitivity determination

The wild-type *Lasiodiplodia theobromae* strain LTTK16-3 used in genomic sequence and genetic transformation was obtained from a diseased Chinese hickory tree (cultivar of Linan) in Linan, Zhejiang province of China. The LTTK16-3 strain and the resulting transformants were stored on the potato dextrose agar (200 g potato, 20 g glucose, and 20 g agar per liter of pure water) slants at 4°C. To determine the sensitivity to G-418, 5-mm mycelial plugs of each strain taken from a 36-h-old colony edge were inoculated on PDA supplemented without/with each stress agent and then incubated at 25°C in the dark. The concentrations for G-418 were indicated in figure legends. Each experiment was repeated three times independently.

### DNA extraction, genome sequencing, and assembly

For the preparation of genomic DNA used in genome sequencing and assembly, the *L. theobromae* strain LTTK16-3 was incubated on PDA plates at 25°C in the dark. After mycelia grew to cover nearly two-thirds of the PDA plate surfaces, the cultures were transferred to a mortar and ground with liquid nitrogen. The resultant powder was placed in a 2-mL centrifuge tube, and the genomic DNA was extracted using a Genomic DNA Kit (Sangon Biotech Co., Ltd., Shanghai) according to the manufacturer’s instructions. After assessing the purity and concentration of the extracted genomic DNA with a NanoDrop One spectrophotometer (Thermo Fisher Scientific, Wilmington, DE, USA), the genomic sequence was analyzed by the Oxford Nanopore Technologies (ONT) PromethION sequencing platform at Biomarker Technologies Co., Ltd (Beijing, China). Additionally, the RNA sequencing was also performed on the Illumina HiSeq4000 sequencing platform using the above mycelium materials collected from PDA media to provide transcription evidence. The related RNA-seq data were deposited in Genome Sequence Archive (GSA) under the accession number CRR332439. The low-quality readings were filtered and high-quality filtered sub-reads were assembled using NextDenovo v2.4.0 and NextPolish v1.3.1 (both available online at https://github.com/Nextomics). A 21-mer was selected for k-mer analysis, and the 21-mer depth frequency distribution was calculated using jellyfish (version 1.1.12). The genome size and heterozygosity were visualized using GenomeScope (version 1.0). The completeness of genome assemblies was assessed using BUSCO v5.12 (https://busco.ezlab.org/). The telomere repeats were determined at the start or end region of contigs (5′-TTAGGG-3′/5′-CCCTAA-3′) to test if contig has reached T2T chromosomal level (36). Repeats were masked before gene prediction by RepeatMasker v4.1.2 (http://www.repeatmasker.org/) using a *de novo* repeat library generated by RepeatModeler v2.01 (http://www.repeatmasker.org/RepeatModeler/). The raw sequence data are publicly available at GSA under the accessions number CRR332440, and the genome assembly is publicly available at the Genome Warehouse (GWH, https://ngdc.cncb.ac.cn/gwh) under the accession number GWHBFSF00000000 and under BioProject PRJCA005744 in National Genomics Data Center, China National Center for Bioinformation (CNCB-NGDC Members and Partners, 2021, https://ngdc.cncb.ac.cn). Also, the corresponding genome sequencing data have been uploaded to the National Center for Biotechnology Information (NCBI) website under the accession number PRJNA1056688.

### Genome annotation

The repeat-masked genome assemblies were used for gene prediction by BRAKER2 (37), which integrated evidence from relevant RNA-seq data and fungal homologous proteins (fungi_odb10, https://busco-data.ezlab.org/v5/data/lineages/). Orthologous gene cluster analysis was conducted using an online web service OrthoVenn2 (https://orthovenn2.bioinfotoolkits.net/home). The gene functional annotation was conducted against databases including PHI-base v4.12 (http://www.phi-base.org/), dbCAN2 (https://bcb.unl.edu/dbCAN2/), Pfam v34.0 (http://pfam.xfam.org/), and EggNOG v5.0 (http://eggnog5.embl.de/). Additionally, secondary metabolite biosynthetic genes were analyzed using antiSMASH v5.2.0 (https://fungismash.secondarymetabolites.org/) (24).

### Phylogenetic analysis

A homologous single-copy gene-based approach was applied to generate a phylogenetic tree of LTTK16-3 genome sequences. The STAG algorithm of OrthoFinder was used to reconstruct a phylogenetic tree based on homologous genes, and the STRIDE algorithm was used to root the species tree in OrthoFinder (25, 26). The genomic data of *Diplodia seriata* (38), *L. theobromae* strain AM2As (12), and *L. theobromae* strain CITRA15 (8) used in phylogenetic analysis were obtained from the NCBI web portal. The genomic data of the other four Chinese hickory trunk cankers associated *Botryosphaeria* species (including *B. dothidea*, *B. fabicerciana*, *B. qingyuanensis*, and *B. cortices*) published in our previous study were obtained from the GWH (https://ngdc.cncb.ac.cn/gwh) (19, 20).

### Construction of recombinant plasmids

The corresponding native promoter and open reading frame of LtActin and LtH1 were amplified using the primer pairs listed in Table S1. For generating the native promoter-LtActin-GFP fusion construct and native promoter-LtH1-GFP fusion construct, the above native promoter-LtActin and promoter-LtH1 fragment were co-transformed with XhoI-digested pYF11 into yeast strain XK125, respectively. The pYF11 contains the neomycin resistance gene, encoding aminoglycoside phosphotransferase, which allows the selection of the resulting fungal transformants using geneticin (G-418), and its map is provided in Fig. S3. The yeast plasmids pYF11-LtActin-GFP and pYF11-LtH1-GFP were rescued from the resulting Trp^+^ yeast transformants. Then, the recombinant plasmids were extracted and transformed into *Escherichia coli* DH5α for large-scale amplification. The resulting recombinant plasmids were verified by sequencing to ensure the accuracy of the in-frame fusion region. The verified recombinant plasmids were selected to construct the fluorescent protein-labeled strains LTTK16-40:: LtActin-GFP and LTTK16-40::LtH1-GFP by transforming these two recombinant plasmids into the protoplasts of the LTTK16-3 strain.

### Fluorescent protein-labeled strain construction

To generate protoplasts, mycelial plugs cut from 36-h-old colony edge were placed into 250-mL flasks containing 100 mL yeast extract peptone dextrose (1% yeast extract, 2% peptone, and 2% glucose) culture. Then, the flasks were kept on a rotary shaker (175 rpm, 25°C) for 16 h, and mycelia were collected using a sterile nylon filter and washed with distilled water thrice to remove medium residue. The harvested mycelia were re-suspended in 10 mL enzymatic digestion solution containing 0.7 M NaCl, 0.3 g cellulase (Ryon Bio-Tech, Shanghai, China, RM1030), 0.3 g lysozyme (Ryon Bio-Tech, Shanghai, China, RM1027), 0.25 g lysing enzyme (Sigma, St. Louis, MO, USA, L1412), and 0.08 g driselase (Sigma-Aldrich, St. Louis, MO, USA, D9515) at 30°C and 85 rpm. The state of protoplasts waThe verified recombinant

s monitored during the enzymatic digestion via microscopy. Protoplasts were separated from cell debris via filtration through three layers of lens-cleaning tissue (2105-802, GE Healthcare, Chicago, IL, USA) and gently rinsed twice with 0.7 M NaCl buffer. Protoplasts were collected via centrifuging at 5,000 rpm at 4°C for 8 min and re-suspended in 5 mL STC buffer [0.8 M sorbitol and 50 mM Tris-HCl (pH 8.0)] twice. For transformation, 750 µL of protoplasts, 200 µL of SPTC (STC with 40%, wt/vol PEG 6000) buffer, 5 µL of heparin (5 mg/mL), and 100 µL (>200 µg/mL) of recombinant plasmids were mixed and incubated on ice for 30 min; then, 400 µL of SPTC was added into the above suspension and incubated at 25°C for 20 min. Transformed protoplasts were mixed into 100 mL RM medium (1 g yeast extract, 1 g casein hydrolysate, 274 g sucrose, and 16 g agar powder per 1 L pure water) at 35°C, poured into petri plates, and incubated at 25°C in dark. After 12 h, RM plates were overlaid with 10 mL of SRM medium (1 g yeast extract, 1 g casein hydrolysate, 342 g sucrose, and 12 g agar per 1 L pure water) containing G-418 for transformant selection and incubated at 25°C. After 2–6 days, geneticin-resistant colonies appeared, and individual transformants were transferred to PDA plates containing G-418 for fluorescence examination. The transformants expressing the GFP-fused protein in right fluorescence localizations were transferred to PDA without G-418 to grow for PCR identification and sequencing. After the PCR identification and sequencing, the right transformants were saved in a PDA slant without G-418 to be used as an inoculation source in the subsequent experiments.

### Microscopic examinations

Fluorescence signals were examined with a Zeiss LSM780 confocal microscope (Gottingen, Niedersachsen, Germany). The subcellular locations of LtActin-GFP and LtH1-GFP were examined using the following confocal microscopy settings: laser 488 nm at 50% power, pinhole 90 µm, and master gain 580.

## Data Availability

The raw sequence data (accession CRR332440) and related RNA-seq data (accession CRR332439) are publicly available at Genome Sequence Archive (GSA, https://ngdc.cncb.ac.cn/gsa/). The genome assembly is publicly available at the Genome Warehouse (GWH, https://ngdc.cncb.ac.cn/gwh) under the accession number GWHBFSF00000000 and under BioProject PRJCA005744 in the National Genomics Data Center, China National Center for Bioinformation (CNCB-NGDC Members and Partners, 2021, https://ngdc.cncb.ac.cn). Also, the corresponding genome sequencing data have been uploaded to the NCBI website under the accession number PRJNA1056688.

## SUPPLEMENTAL MATERIAL

Supplemental Materials spectrum.03311-23-s0001.docx Table S1, captions for Tables S2 to S5, and Figures S1 to S3.

Table S2 spectrum.03311-23-s0002.xlsx KEGG analysis of 8,818 core orthologous protein clusters of five Botryosphaeriaceae species.

Table S3 spectrum.03311-23-s0003.xlsx Bidirectional BLAST analysis of LTTK16-3 strain specific proteins in the other four Chinese hickory trunk canker related *Botryosphaeria* strains.

Table S4 spectrum.03311-23-s0004.xlsx GO annotation of LTTK16-3 stain specific proteins.

Table S5 spectrum.03311-23-s0005.xlsx Pfam domain annotation of LTTK16-3 stain specific proteins.

ASM does not own the copyrights to Supplemental Material that may be linked to, or accessed through, an article. The authors have granted ASM a non-exclusive, world-wide license to publish the Supplemental Material files. Please contact the corresponding author directly for reuse.

## References

[1] Dai DJ, Wang HD, Wang YP, Zhang CQ. 2017. Management of Chinese hickory (Carya cathayensis) trunk canker through effective fungicide application programs and baseline sensitivity of Botryosphaeria dothidea to trifloxystrobin. Australasian Plant Pathol 46:75–82. doi:10.1007/s13313-017-0465-4

[2] Zhu C, Deng X, Shi F. 2008. Evaluation of the antioxidant activity of Chinese hickory Carya cathayensis kernel ethanol extraction. African J Biotechnol 7:2169–2173. doi:10.5897/AJB08.344

[3] Wang QW, Zhang CQ. 2019. Q-LAMP assays for the detection of Botryosphaeria dothidea causing Chinese hickory canker in trunk, water, and air samples. Plant Dis 103:3142–3149. doi:10.1094/PDIS-04-19-0773-RE 31560617

[4] Zhang C, Xu B. 2012. First report of canker on Chinese hickory (Carya cathayensis) caused by Botryosphaeria dothidea in China. Plant Dis 95:1319–1319. doi:10.1094/PDIS-05-11-0457 30731659

[5] Yang Y, Huang Q, Wang X, Mei J, Sharma A, Tripathi DK, Yuan H, Zheng B. 2021. Genome-wide identification and expression profiles of ABCB gene family in Chinese hickory (Carya cathayensis Sarg.) during grafting. Plant Physiol Biochem 168:477–487. doi:10.1016/j.plaphy.2021.10.029 34757298

[6] Zhuang CJ, Wang QW, Wu QQ, Qiu ZL, Xu BC, Zhang CQ. 2021. Diversity of Botryosphaeriaceae species associated with Chinese hickory tree (Carya cathayensis) trunk cankers. Plant Dis 105:3869–3879. doi:10.1094/PDIS-02-21-0289-RE 34213972

[7] Ma T, Zhang Y, Yan C, Zhang C. 2023. Phenotypic and genomic difference among four Botryosphaeria pathogens in Chinese hickory trunk canker. J Fungi (Basel) 9:204. doi:10.3390/jof9020204 36836318 PMC9963396

[8] Zheng Q, Ozbudak E, Liu G, Hosmani PS, Saha S, Flores-Gonzalez M, Mueller LA, Rodrigues-Stuart K, Dewdney MM, Lin Y, Zhang J, Tarazona YC, Liu B, Oliva R, Ritenour MA, Cano LM. 2021. Draft genome sequence resource of the citrus stem-end rot fungal pathogen Lasiodiplodia theobromae CITRA15. Phytopathology 111:761–764. doi:10.1094/PHYTO-08-20-0349-A 33190608

[9] Zaher AM, Moharram AM, Davis R, Panizzi P, Makboul MA, Calderón AI. 2015. Characterisation of the metabolites of an antibacterial endophyte Botryodiplodia theobromae Pat. of Dracaena draco L. by LC–MS/MS. Nat Prod Res 29:2275–2281. doi:10.1080/14786419.2015.1012715 25693860

[10] Li Z, Wang Y-T, Gao L, Wang F, Ye J-L, Li G-H. 2014. Biochemical changes and defence responses during the development of peach gummosis caused by Lasiodiplodia theobromae. Eur J Plant Pathol 138:195–207. doi:10.1007/s10658-013-0322-4

[11] Wang F, Zhao L, Li G, Huang J, Hsiang T. 2011. Identification and characterization of Botryosphaeria spp. causing gummosis of peach trees in Hubei province, central China. Plant Dis 95:1378–1384. doi:10.1094/PDIS-12-10-0893 30731783

[12] Ali SS, Asman A, Shao J, Balidion JF, Strem MD, Puig AS, Meinhardt LW, Bailey BA. 2020. Genome and transcriptome analysis of the latent pathogen Lasiodiplodia theobromae, an emerging threat to the cacao industry. Genome 63:37–52. doi:10.1139/gen-2019-0112 31580730

[13] Ho A, Di Lonardo DP, Bodelier PLE. 2017. Revisiting life strategy concepts in environmental microbial ecology. FEMS Microbiol Ecol 93. doi:10.1093/femsec/fix006 28115400

[14] Bochner BR, Gadzinski P, Panomitros E. 2001. Phenotype microarrays for high-throughput phenotypic testing and assay of gene function. Genome Res 11:1246–1255. doi:10.1101/gr.186501 11435407 PMC311101

[15] Bochner BR. 2003. New technologies to assess genotype-phenotype relationships. Nat Rev Genet 4:309–314. doi:10.1038/nrg1046 12671661

[16] Zhan S, Fang G, Cai M, Kou Z, Xu J, Cao Y, Bai L, Zhang Y, Jiang Y, Luo X, Xu J, Xu X, Zheng L, Yu Z, Yang H, Zhang Z, Wang S, Tomberlin JK, Zhang J, Huang Y. 2020. Genomic landscape and genetic manipulation of the black soldier fly Hermetia illucens, a natural waste recycler. Cell Res 30:50–60. doi:10.1038/s41422-019-0252-6 31767972 PMC6951338

[17] Lan T, Li H, Yang S, Shi M, Han L, Sahu SK, Lu Y, Wang J, Zhou M, Liu H, Huang J, Wang Q, Zhu Y, Wang L, Xu Y, Lin C, Liu H, Hou Z. 2022. The chromosome-scale genome of the raccoon dog: Insights into its evolutionary characteristics. iScience 25:105117. doi:10.1016/j.isci.2022.105117 36185367 PMC9523411

[18] Li D, Tang Y, Lin J, Cai W. 2017. Methods for genetic transformation of filamentous fungi. Microb Cell Fact 16:168. doi:10.1186/s12934-017-0785-7 28974205 PMC5627406

[19] Bao J, Wu Q, Huang J, Zhang C-Q. 2022. High-quality genome assembly and annotation resource of Botryosphaeria dothidea strain BDLA16-7, causing trunk canker disease on Chinese hickory. Plant Dis 106:1023–1026. doi:10.1094/PDIS-08-21-1623-A 34735279

[20] Wu Q, Liu Y, Hu S, Huang J, Zhang C. 2022. High-quality genome assembly and annotation resource of three Botryosphaeria pathogens causing Chinese Hickory canker. Mol Plant Microbe Interact 35:941–943. doi:10.1094/MPMI-03-22-0055-A 35724311

[21] Muniz CR, da Silva GF, Souza MT Jr, Freire FCO, Kema GHJ, Guedes MIF. 2014. Agrobacterium tumefaciens-mediated transformation of Lasiodiplodia theobromae, the causal agent of gummosis in cashew nut plants. Genet Mol Res 13:2906–2913. doi:10.4238/2014.February.21.8 24634294

[22] Liu Y, Zhang Z, Fu J, Wang G, Wang J, Liu Y. 2017. Transcriptome analysis of maize immature embryos reveals the roles of cysteine in improving Agrobacterium infection efficiency. Front Plant Sci 8:1778. doi:10.3389/fpls.2017.01778 29089955 PMC5651077

[23] Bao J, Chen M, Zhong Z, Tang W, Lin L, Zhang X, Jiang H, Zhang D, Miao C, Tang H, Zhang J, Lu G, Ming R, Norvienyeku J, Wang B, Wang Z. 2017. Pacbio sequencing reveals transposable elements as a key contributor to genomic plasticity and virulence variation in Magnaporthe oryzae. Mol Plant 10:1465–1468. doi:10.1016/j.molp.2017.08.008 28838703

[24] Blin K, Shaw S, Steinke K, Villebro R, Ziemert N, Lee SY, Medema MH, Weber T. 2019. AntiSMASH 5.0: updates to the secondary metabolite genome mining pipeline. Nucleic Acids Res 47:W81–W87. doi:10.1093/nar/gkz310 31032519 PMC6602434

[25] Emms DM, Kelly S. 2017. STRIDE: species tree root inference from gene duplication events. Mol Biol Evol 34:3267–3278. doi:10.1093/molbev/msx259 29029342 PMC5850722

[26] Emms DM, Kelly S. 2019. OrthoFinder: phylogenetic orthology inference for comparative genomics. Genome Biol 20:238. doi:10.1186/s13059-019-1832-y 31727128 PMC6857279

[27] Miranda-Saavedra D, Stark MJR, Packer JC, Vivares CP, Doerig C, Barton GJ. 2007. The complement of protein kinases of the microsporidium Encephalitozoon cuniculi in relation to those of Saccharomyces cerevisiae and Schizosaccharomyces pombe. BMC Genomics 8:309. doi:10.1186/1471-2164-8-309 17784954 PMC2078597

[28] Wang C, Zhang S, Hou R, Zhao Z, Zheng Q, Xu Q, Zheng D, Wang G, Liu H, Gao X, Ma J-W, Kistler HC, Kang Z, Xu J-R, Howlett BJ. 2011. Functional analysis of the kinome of the wheat scab fungus Fusarium graminearum. PLoS Pathog 7:e1002460. doi:10.1371/journal.ppat.1002460 22216007 PMC3245316

[29] Liu N, Yun Y, Yin Y, Hahn M, Ma Z, Chen Y. 2019. Lipid droplet biogenesis regulated by the FgNem1/Spo7-FgPah1 phosphatase cascade plays critical roles in fungal development and virulence in Fusarium graminearum. New Phytol 223:412–429. doi:10.1111/nph.15748 30767239

[30] Yin Y, Wu S, Chui C, Ma T, Jiang H, Hahn M, Ma Z, Wang Y. 2018. The MAPK kinase BcMkk1 suppresses oxalic acid biosynthesis via impeding phosphorylation of BcRim15 by BcSch9 in Botrytis cinerea. PLoS Pathog 14:e1007285. doi:10.1371/journal.ppat.1007285 30212570 PMC6136818

[31] Ianiri G, Averette AF, Kingsbury JM, Heitman J, Idnurm A. 2016. Gene function analysis in the ubiquitous human commensal and pathogen Malassezia genus. mBio 7:e01853-16. doi:10.1128/mBio.01853-16 27899504 PMC5137500

[32] Liu H, Zhao H, Wu L, Xu W. 2017. A genetic transformation method for cadmium hyperaccumulator Sedum plumbizincicola and non-hyperaccumulating ccotype of Sedum alfredii. Front Plant Sci 8:1047. doi:10.3389/fpls.2017.01047 28670322 PMC5472854

[33] Kim J-A, Kim J-M, Kim H-G, Kim B-T, Hwang K-J, Park S-M, Yang M-S, Kim D-H. 2009. Protoplast-mediated transformation of the filamentous fungus Cladosporium phlei: evidence of tandem repeats of the integrative transforming vector. Plant Pathol J 25:179–183. doi:10.5423/PPJ.2009.25.2.179

[34] Men P, Wang M, Li J, Geng C, Huang X, Lu X. 2021. Establishing an efficient genetic manipulation system for sulfated echinocandin producing fungus Coleophoma empetri. Front Microbiol 12:734780. doi:10.3389/fmicb.2021.734780 34489920 PMC8417879

[35] Becker DM, Lundblad V. 2001. Introduction of DNA into yeast cells. Curr Protoc Mol Biol Chapter 13:Unit13. doi:10.1002/0471142727.mb1307s27 18265102

[36] Miga KH, Koren S, Rhie A, Vollger MR, Gershman A, Bzikadze A, Brooks S, Howe E, Porubsky D, Logsdon GA, et al.. 2020. Telomere-to-telomere assembly of a complete human X chromosome. Nature 585:79–84. doi:10.1038/s41586-020-2547-7 32663838 PMC7484160

[37] Brůna T, Hoff KJ, Lomsadze A, Stanke M, Borodovsky M. 2021. BRAKER2: automatic eukaryotic genome annotation with GeneMark-EP+ and AUGUSTUS supported by a protein database. NAR Genom Bioinform 3:lqaa108. doi:10.1093/nargab/lqaa108 33575650 PMC7787252

[38] Robert-Siegwald G, Vallet J, Abou-Mansour E, Xu J, Rey P, Bertsch C, Rego C, Larignon P, Fontaine F, Lebrun MH. 2017. Draft genome sequence of Diplodia seriata F98.1, a fungal species involved in grapevine trunk diseases. Genome Announc 5:e00061-17. doi:10.1128/genomeA.00061-17 28385831 PMC5383879

