# Supplementary material for: High-quality genome assembly and genetic transformation system of Lasiodiplodia theobromae strain LTTK16-3, a fungal pathogen of Chinese hickory
Source: Microbiol Spectr. 2024 Feb 13;12(3):e03311-23. doi: 10.1128/spectrum.03311-23 (PMC10913528; doi:10.1128/spectrum.03311-23)
Supplement: Supplemental Materials — Table S1, captions for Tables S2 to S5, and Figures S1 to S3. [file spectrum.03311-23-s0001.docx]

**Supplemental Materials**

**Supplementary Table S1** Primers used in this study and their relevant characteristics.

| **Primer** | **Sequence(5’-3’)** | **Relevant Characteristics** |
| --- | --- | --- |
| Actin-GFP-F | ACTCACTATAGGGCGAATTGGGTACTCAAATTGGTTCGACAGCACTGCCGACGCTG | PCR primers to amplify *LtActin* fragment used for construction of the LtActin-GFP vector |
| Actin-GFP-R | CACCACCCCGGTGAACAGCTCCTCGCCCTTGCTCACGAAGCACTTGCGGTGGACAATG |  |
| Actin-GFP-ID-F | GGTCGTGATCTTACCGACTACC | PCR primers for the identification of the in-frame LtActin-GFP fusion vector from yeast |
| Actin-GFP-ID-R | GACACGCTGAACTTGTGGCCGTT |  |
| H1-GFP-F | ACTCACTATAGGGCGAATTGGGTACTCAAATTGGTTGAGACGGGTTGCCCGACTTC | PCR primers to amplify *LtH1* fragment used for construction of the LtH1-GFP vector |
| H1-GFP-R | CACCACCCCGGTGAACAGCTCCTCGCCCTTGCTCACAGCCTTGGTATCAGCCTCGGC |  |
| H1-GFP-ID-F | GCTGACACGACTGTACAGCCG | PCR primers for the identification of the in-frame LtH1-GFP fusion vector from yeast |
| H1-GFP-ID-R | GACACGCTGAACTTGTGGCCGTT |  |

**Supplementary Table S2** The Kyoto Encyclopedia of Genes and Genomes (KEGG) analysis of 8818 core orthologous protein clusters of five *Botryosphaeriaceae* species.

**Supplementary Table S3** Bidirectional BLAST analysis of LTTK16-3 stain specific proteins in the other four Chinese hickory trunk canker related *Botryosphaeria* strains

**Supplementary Table S4** GO annotation of LTTK16-3 stain specific proteins.

**Supplementary Table S5** Pfam domain annotation of LTTK16-3 stain specific proteins.

**
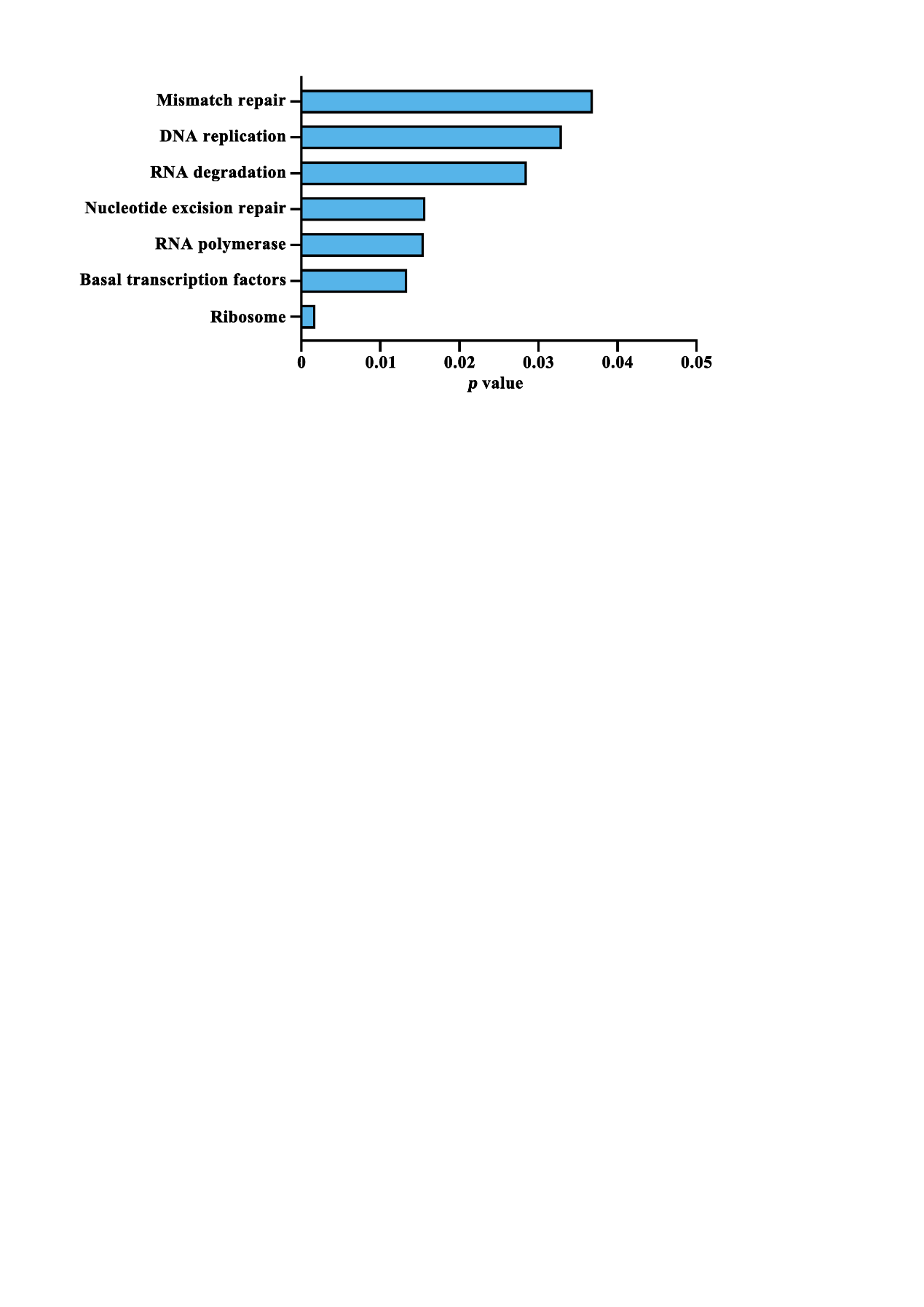
**

**Supplementary Figure S1** Enrichment of five *Botryosphaeriaceae* species core orthologous protein clusters on the KEGG pathways (p < 0.05).


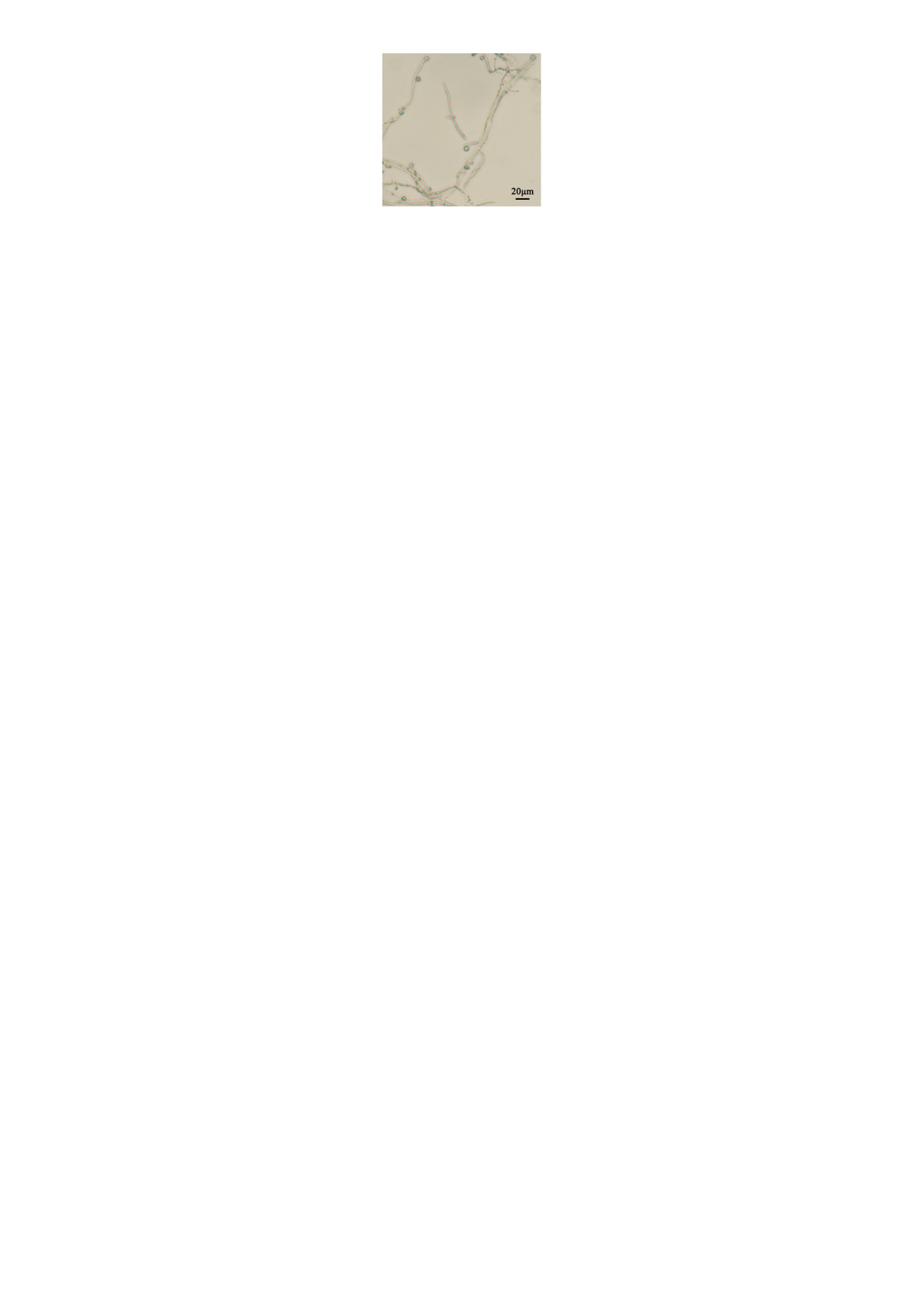


**Supplementary Figure S2** The microscope examination showing protoplasts begin to produce after digested at 30 ℃ for 0.5 h. Bars: 20 µm.


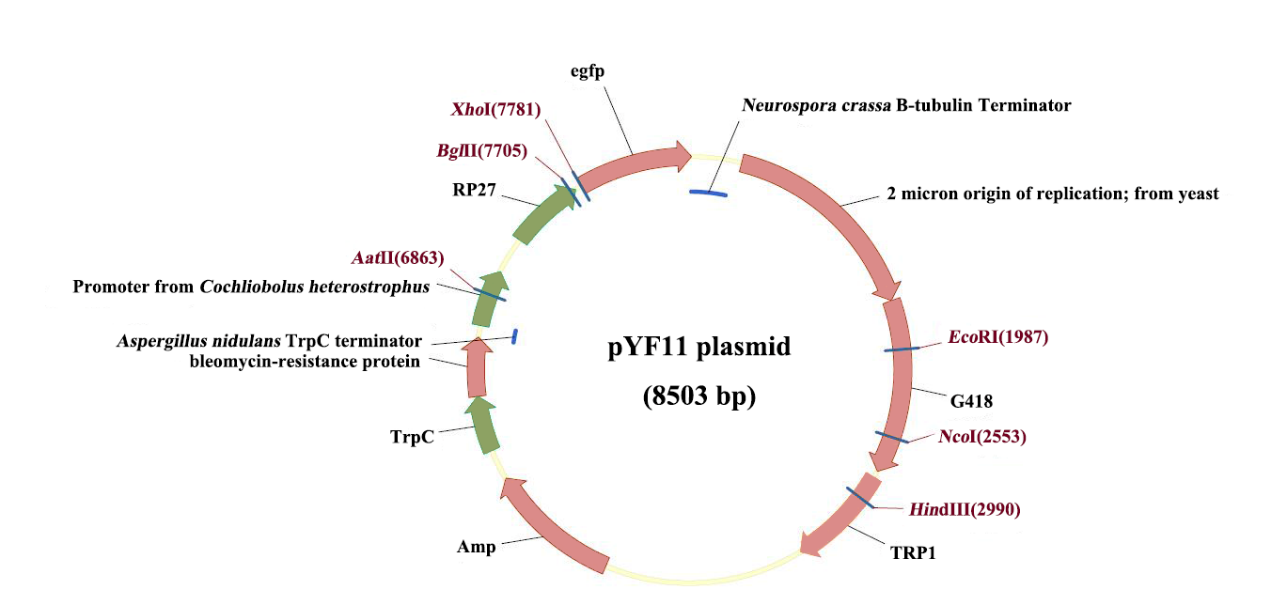


**Supplementary Figure S3** The vector map of the plasmid pYF11.
